# Herpes simplex virus type 1 inflammasome activation in proinflammatory human macrophages is dependent on NLRP3, ASC, and caspase-1

**DOI:** 10.1371/journal.pone.0229570

**Published:** 2020-02-26

**Authors:** Andrew H. Karaba, Alexis Figueroa, Guido Massaccesi, Sara Botto, Victor R. DeFilippis, Andrea L. Cox

**Affiliations:** 1 Division of Infectious Diseases, Department of Medicine, Johns Hopkins University School of Medicine, Baltimore, MD, United States of America; 2 Vaccine and Gene Therapy Institute, Oregon Health and Science University, Portland, OR, United States of America; Rowan University, UNITED STATES

## Abstract

The proinflammatory cytokines interleukin (IL)-1β and IL-18 are products of activation of the inflammasome, an innate sensing system, and important in the pathogenesis of herpes simplex virus type 1 (HSV-1). The release of IL-18 and IL-1β from monocytes/macrophages is critical for protection from HSV-1 based on animal models of encephalitis and genital infection, yet if and how HSV-1 activates inflammasomes in human macrophages is unknown. To investigate this, we utilized both primary human monocyte derived macrophages and human monocytic cell lines (THP-1 cells) with various inflammasome components knocked-out. We found that HSV-1 activates inflammasome signaling in proinflammatory primary human macrophages, but not in resting macrophages. Additionally, HSV-1 inflammasome activation in THP-1 cells is dependent on nucleotide-binding domain and leucine-rich repeat-containing receptor 3 (NLRP3), apoptosis-associated speck-like molecule containing a caspase recruitment domain (ASC), and caspase-1, but not on absent in melanoma 2 (AIM2), or gamma interferon-inducible protein 16 (IFI16). In contrast, HSV-1 activates non-canonical inflammasome signaling in proinflammatory macrophages that results in IL-1β, but not IL-18, release that is independent of NLRP3, ASC, and caspase-1. Ultraviolet irradiation of HSV-1 enhanced inflammasome activation, demonstrating that viral replication suppresses inflammasome activation. These results confirm that HSV-1 is capable of activating the inflammasome in human macrophages through an NLRP3 dependent process and that the virus has evolved an NLRP3 specific mechanism to inhibit inflammasome activation in macrophages.

## Introduction

The ability to quickly recognize and respond to pathogens is essential to host survival. The first opportunity to do so lies in the innate immune response. One of the most essential aspects of this response is the recognition of pathogen associated molecular patterns (PAMPs) on the invading pathogen by the pattern recognition receptors (PRRs) of host cells [1]. This interaction leads to a number of molecular and cellular signals that serve to protect the host on cellular and organism levels. One such innate signaling system is the formation of inflammasomes, which are intracellular multi-protein complexes that regulate an inflammatory type of cell death called pyroptosis as well as the production of mature forms of the inflammatory cytokines IL-1β and IL-18 [2]. Macrophages and myeloid dendritic cells (mDCs) are the primary producers of these potent proinflammatory cytokines, which drive type 1 immunity in natural killer cells and T cells [3]. The production of these cytokines requires two steps. The first step, sometimes referred to as priming, requires activation of the nuclear factor κB (NF-κB) pathway through the recognition of a PAMP leading to synthesis of components of the inflammasome, including pro-IL-1β, pro-IL-18, and pro-caspase-1. The second step involves PRR activation, oligomerization, and assembly of the inflammasome. This takes place through one of multiple receptor or adapter proteins that recognize various PAMPs or danger-associated molecular patterns (DAMPs). These include members of the nucleotide-binding domain and leucine-rich repeat-containing receptors (NLR) family of proteins, absent in melanoma 2 (AIM2), and pyrin. NLRP3 responds to a diverse group of PAMPs and DAMPs, particularly viral RNA [4–7]. In contrast, AIM2 is activated after binding to cytoplasmic double stranded DNA (dsDNA) [8]. Recognition of an appropriate PAMP or DAMP by one of these adapter proteins leads to apoptosis-associated speck-like molecule containing a caspase recruitment domain (ASC) assembly and oligomerization followed by pro-caspase-1 recruitment to the complex. Pro-caspase-1 autocatalysis to active caspase-1 allows for cleavage of pro-IL-1β and pro-IL-18 to their active forms, IL-1β and IL-18, and then secretion into the extracellular space (reviewed in [2,9,10]). There are other “non-canonical” sensors and caspases that can lead to inflammasome cytokine release, but the caspase-1 pathway is thought to be the most relevant in viral infection [11,12].

A number of viruses are known to activate the inflammasome, including influenza, hepatitis C (HCV), HIV, and herpesviruses [10,13]. Herpes simplex virus type 1 (HSV-1) is a neurotropic alphaherpesvirus that predominantly infects epithelial cells and neurons, but has broad cell tropism [14]. Specifically, it can infect macrophages, which are one of the predominant cell types that infiltrate the eye after corneal infection and are crucial to the innate immune response to HSV-1 and other viruses [15–17]. Furthermore, monocyte/macrophage production of IL-1β and IL-18 is critical to prevent severe HSV disease in encephalitis, keratitis, and vaginal infection in mouse models [17–20]. Therefore, understanding how HSV-1 activates the inflammasome in these cells is key to developing a comprehensive view of HSV-1 pathogenesis. A previous study demonstrated that the HSV-1 viral tegument protein VP22 specifically blocks AIM2 inflammasome activation and signaling in the THP-1 monocyte/macrophage cell line despite production of IL-1β, leaving the mechanism of HSV-1 activation of the inflammasome in these cells to be defined [21,22]. Thus, it remains unclear which adapters are required for HSV-1 induction of inflammasome activation in macrophages.

Here, we report that HSV-1 activates canonical inflammasome signaling, as measured by IL-18, in both proinflammatory primary human macrophages and THP-1 cells. Additionally, this activation requires NLRP3, ASC, and caspase-1, but not AIM2 or IFI16.

## Results

### HSV-1 activates the inflammasome in primary human monocyte derived macrophages

**Fig 1:**
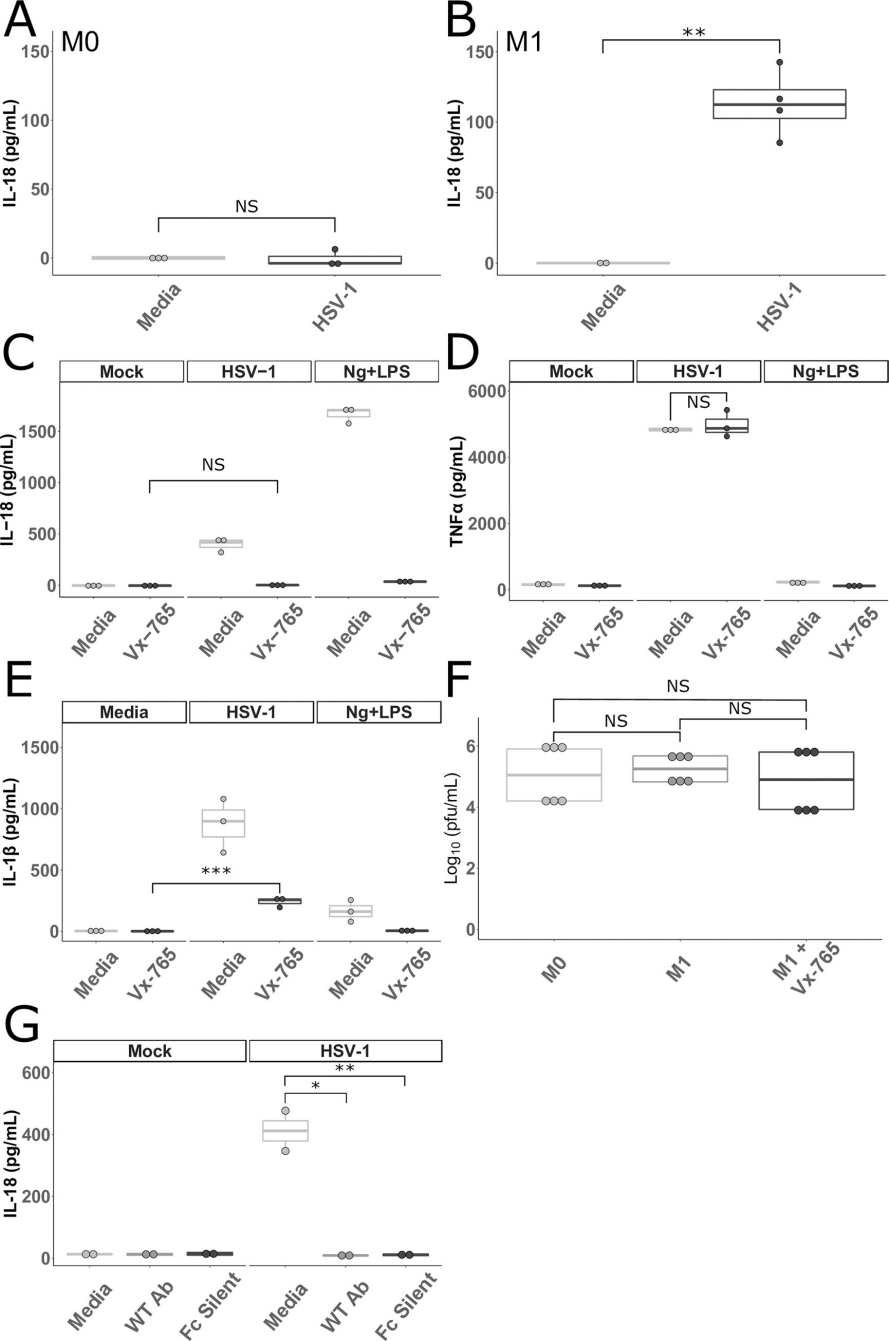
HSV-1 activates inflammasomes in primary human macrophages. **A and B.** Primary human MDMs cultured without (M0 **A**) or with IFNγ (M1 **B**) were incubated with HSV-1 or media for 24 hours. Cell culture supernatants were collected and assayed for IL-18 (**B** represents the combination of two experiments). **C, D, and E.** Primary human MDMs stimulated with IFNγ were cultured in media alone or in media containing 100 μg/mL of VX-765 (Invivogen, San Diego, California) and then incubated with HSV-1, nigericin and LPS (Ng+LPS), or media for 24 hours as outlined in Materials and Methods. Cell culture supernatants were collected and assayed for (**C**) IL-18, (**D**) TNFα, and (**E**) IL-1β. **F.** MDMs cultured without IFNγ (M0), with IFNγ (M1), or with IFNγ and VX-765 (M1+Vx-765) were infected with HSV-1 for 1 hour followed by citrate wash to inactivate any extracellular virus. Supernatants were collected 24 hours later and plaque forming units (PFU) were determined via standard plaque assay on Vero cells. Data shown are combined from two independent experiments. **G.** MDMs stimulated with IFNγ (M1) were incubated with HSV-1 or media as well as a neutralizing antibody (WT Ab) and a neutralizing antibody unable to bind to Fc receptors (Fc Silent) for 24 hours. Cell culture supernatants were collected and assayed for IL-18. Differences between groups indicated by brackets were determined by a Student’s *t*-test. NS, *,**,*** indicate p-values >0.05, <0.05, <0.01, <0.001, respectively. The lower and upper borders of the boxplots represent the 1^st^ quartile and 3^rd^ quartile respectively. The median is represented by a horizontal line in the box. The lower and upper whiskers represent 1.5x the interquartile range (IQR) beyond the quartile lines. Each dot represents an individual sample.

HSV-1 is known to activate the inflammasome in THP-1 cells, but the mechanism is unknown and it has not been studied extensively in primary human macrophages [21,22]. To determine if HSV-1 is capable of activating the inflammasome in primary human monocyte derived macrophages (MDMs) we infected resting macrophages (referred to as M0) with HSV-1 and measured IL-18 in supernatants 24 hours later. The amount of IL-18 detected in HSV-1 infected M0 macrophages was not different than the amount detected in mock infected macrophages (**Fig 1A**). However, some viruses, such as Dengue virus, require a more inflammatory macrophage phenotype to induce inflammasome activation [23]. Therefore, we incubated MDMs with IFNγ (referred to as M1) prior to infection with HSV-1 to induce a more proinflammatory cell [24,25]. Unlike the M0 macrophages, M1 macrophages did produce significant amounts of IL-18 after HSV-1 infection (**Fig 1B**). Both M0 and M1 macrophages produced IL-18 after incubation with nigericin (Ng) and LPS, a potent activator of the NLRP3 inflammasome [26,27] (**S1A Fig**). Proinflammatory (M1) macrophages are often produced by incubating MDMs with IFNγ and LPS [28,29]. However, we found no difference in the amount of IL-18 produced after HSV-1 infection by M1s stimulated with IFNγ or IFNγ and LPS (**S1B Fig**). Therefore, we opted to omit the LPS in subsequent experiments.

While mature forms of both IL-18 and IL-1β are produced by canonical caspase-1 inflammasome formation, other caspases are capable of processing IL-18 and IL-1β (reviewed in [30]). These “non-canonical” pathways do not require an adapter molecule and are well described for IL-1β. However, mature IL-18 production is thought to be restricted to canonical inflammasome formation aside from very limited circumstances (reviewed in [31,32]). To determine if the production of IL-18 by macrophages after HSV-1 infection was due to canonical inflammasome activation, M1 MDMs were treated with VX-765, a caspase-1 specific inhibitor [33–35], prior to either infection with HSV-1 or treatment with nigericin and LPS. IL-18 in cell supernatants was reduced to amounts not significantly different from background in the presence of VX-765, suggesting that IL-18 production is due to canonical inflammasome activation (**Fig 1C**). To ensure that VX-765 was neither toxic to the cells nor non-specifically interfering with HSV-1 sensing by the macrophages, the same supernatants were tested for TNFα, which is produced by macrophages in response to HSV-1 infection [36]. There was no significant difference in the amount of TNFα produced after HSV-1 infection of macrophages incubated with or without VX-765 (**Fig 1D**). Interestingly, when IL-1β was measured from these same supernatants, treatment with VX-765 did not reduce IL-1β levels to background levels in the HSV-1 infected macrophages, but did in the Ng+LPS treated macrophages (**Fig 1E**). This suggests that HSV-1 induces IL-1β release by both caspase-1 dependent and independent mechanisms, and that IL-18 is a more specific marker of canonical inflammasome activation in this system.

To ensure that the varying amounts of IL-18 observed in these experiments was not due to changes in the ability of HSV-1 to replicate in macrophages exposed to IFNγ and/or VX-765, M0 and M1 MDMs (with and without VX-765) were infected with HSV-1. Culture supernatants were collected after 24 hours, and plaque forming units (PFU) were determined using a standard plaque assay. No differences were observed in HSV-1 replication in macrophages exposed to IFNγ or VX-765 (**Fig 1F**). These results demonstrate that viral replication alone does not account for differences in inflammasome activation in primary human macrophages.

HIV and HCV are capable of activating the inflammasome in monocytes/macrophages via clathrin mediated endocytosis without infecting them or binding known viral entry receptors [13]. To test whether HSV-1 activation of the inflammasome in macrophages requires entry and infection through known mechanisms, MDMs pretreated with IFNγ were inoculated with HSV-1 mixed with neutralizing human monoclonal antibodies directed against the HSV-1 glycoprotein D, which is required for HSV-1 entry [37]. Both WT antibodies and antibodies unable to bind to Fc receptors (Fc Silent) were utilized to control for any Fc-mediated entry of virus. While HSV-1 alone induced robust IL-18 release, this was reduced to background levels in the presence of neutralizing antibody regardless of whether the antibody was capable of binding to Fc receptors (**Fig 1G**). These data confirm that unlike HCV and HIV, HSV-1 must enter macrophages via viral glycoprotein and receptor mediated pathways to induce inflammasome activation.

### NLRP3, ASC, and caspase-1 are required for inflammasome activation in response to HSV-1

**Fig 2:**
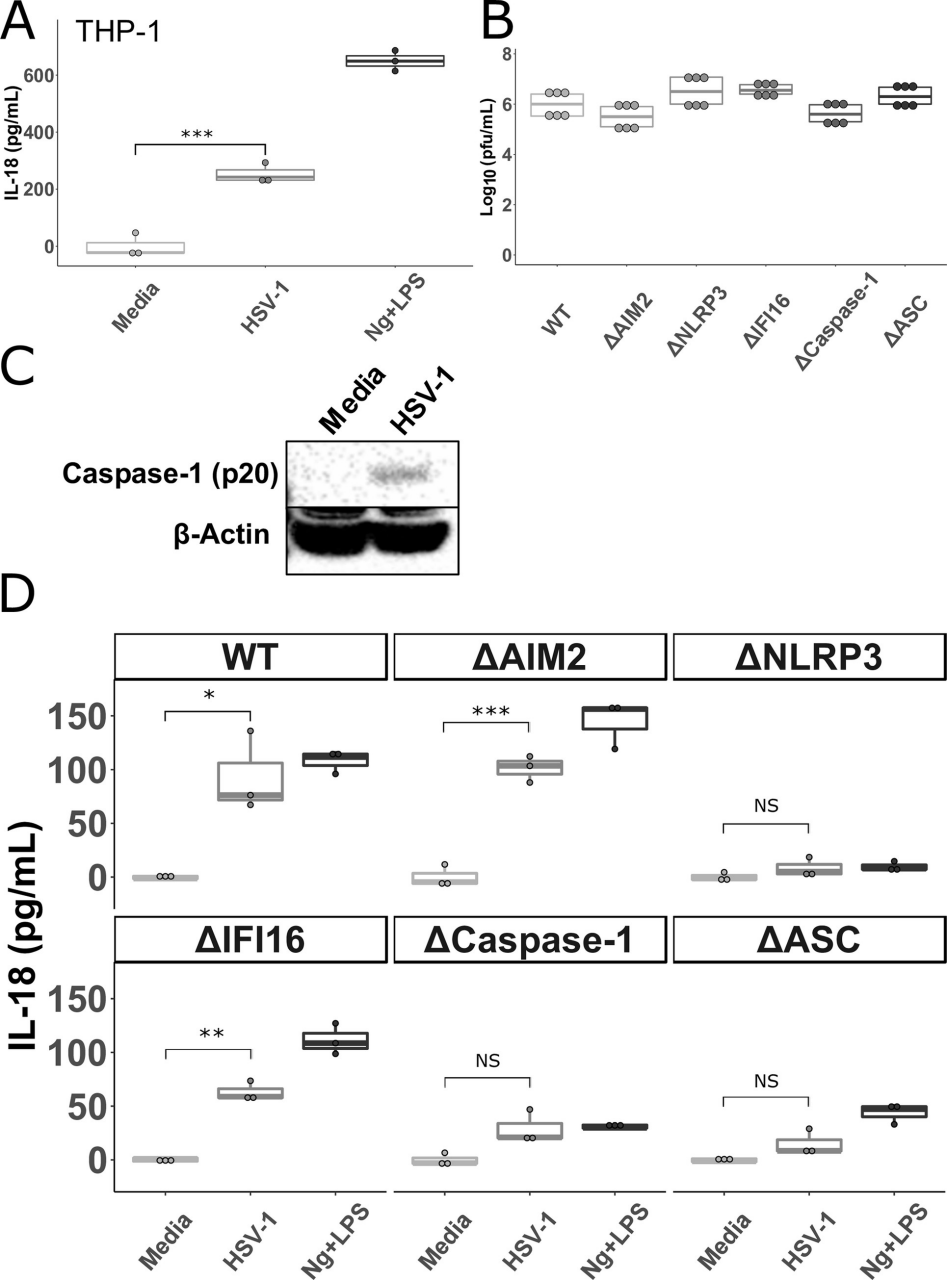
HSV-1 inflammasome activation in THP-1 cells is dependent on NLRP3, ASC, and caspase-1. **A.** THP-1 cells were stimulated overnight with PMA (100 ng/mL) and then incubated with HSV-1, nigericin and LPS (Ng+LPS), or media, as outlined in Materials and Methods, for 24 hours. Cell culture supernatants were collected and IL-18 was measured via ELISA. **B.** THP-1 cells with the indicated gene disrupted via CRISPR-cas9 (Δ) were stimulated overnight with PMA, infected with HSV-1 for 1 hour followed by citrate wash to inactivate any extracellular virus. Supernatants were collected 24 hours later and PFU were determined via standard plaque assay on Vero cells. Data shown are combined from two independent experiments. **C.** Cell lysates from THP-1 cells infected with HSV-1 or mock infected were probed for caspase-1 or β-actin via western blot. **D.** THP-1 cells with the indicated gene disrupted via CRISPR-cas9 (Δ) were stimulated overnight with PMA and then incubated with HSV-1, nigericin and LPS (Ng+LPS), or media for 24 hours before IL-18 was measured in cell supernatants. ΔHUMCYC cells are labeled as “WT.” Differences between groups indicated by brackets were determined by a Student’s *t*-test. NS, *,**,*** indicate p-values >0.05, <0.05, <0.01, <0.001, respectively.

To confirm that HSV-1 is capable of activating the inflammasome in a monocyte/macrophage cell line so that dependence on specific inflammasome proteins could be assessed, THP-1 cells were primed with phorbol 12-myristate 13-acetate (PMA) overnight and then infected with HSV-1. IL-18 was measured in supernatants after 24 hours. As previously reported [38], THP-1 cells produced IL-18 after infection with HSV-1 (**Fig 2A**). Autoprocessing of caspase-1 results in release of the large (p20) subunit [39]. Therefore, lysates from THP-1 cells either infected with HSV-1 or mock infected for 4 hours were probed for this cleavage product. As expected, the caspase-1 p20 subunit was detected in lysates from HSV-1 infected cells, but not mock infected cells (**Fig 2C**).

Studies in keratinocytes and human foreskin fibroblasts (HFF) found roles for IFI16, NLRP3, and AIM2 in HSV-1 inflammasome activation [40,41]. Yet, monocytes/macrophages produce the majority of inflammasome related cytokines (IL-18 and IL-1β) in other viral infections and play crucial roles in preventing the most severe manifestations of HSV infection in mouse models [13,42]. Therefore, to determine what inflammasome components are required for HSV-1 induced inflammasome activation in macrophages, we infected THP-1 cells lacking various inflammasome proteins. These cells were constructed using the CRISPR-Cas9 system and previously used to determine the requirements for human cytomegalovirus (HCMV) inflammasome activation in macrophages [43]. The ΔHUMCYC cell-line (WT) was used to control for any off-target effects of the CRISPR-cas9 system. This line was derived from the same THP-1 cells, but targeted a human pseudogene (*HUMCYCPS3*). While HSV-1 infection of the WT, ΔAIM2, and ΔIFI16 THP-1 cells led to significant IL-18 production, infection of ΔNLRP3, Δcaspase-1, and ΔASC cells resulted in levels of IL-18 that were not significantly different from mock infection (**Fig 2D**). The amount of IL-18 produced in response to HSV-1 by the ΔAIM2, and ΔIFI16 THP-1 cells was not significantly different from WT (**S2A Fig**). The combination of nigericin with LPS was used as a positive control. As expected, IL-18 concentrations in supernatants from cells lacking NLRP3, ASC, and caspase-1 were not above background after nigericin and LPS exposure [27]. These results indicate that HSV-1 induced canonical inflammasome activation and IL-18 production and release in macrophages is dependent on ASC, caspase-1, and NLRP3, but not on the dsDNA sensors IFI16 or AIM2. These differences were not due to differing replication capacity in the different knock-out lines as HSV-1 replicated similarly in each cell line compared to the WT line (**Fig 2B**).

### UV-irradiated HSV-1 increases inflammasome activation

**Fig 3:**
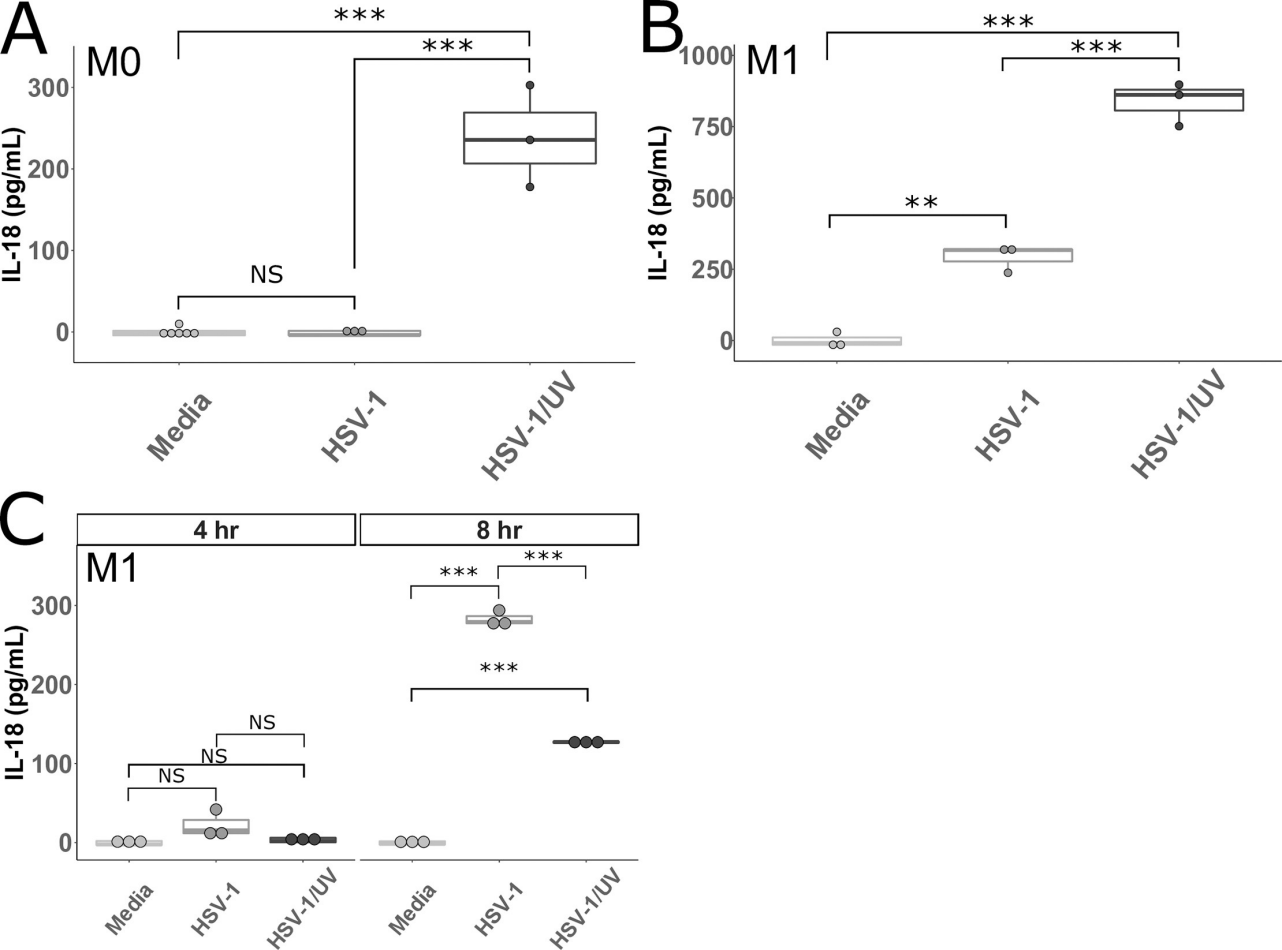
IL-18 produced by MDMs after infection with UV-irradiated HSV-1. **A and B.** Primary human MDMs cultured without (M0 **A**) or with IFNγ (M1 **B**) were incubated with HSV-1, UV irradiated HSV-1 (HSV-1/UV), or media for 24 hours. Cell culture supernatants were collected and assayed for IL-18. **C.** Primary human MDMs cultured with IFNγ were incubated with HSV-1, UV irradiated HSV-1 (HSV-1/UV), or media for 4 hours and 8 hours. Cell culture supernatants were collected and assayed for IL-18. Differences between indicated conditions within a cell type were determined by a one-way ANOVA with Tukey HSD post-hoc analysis. NS, *,**,*** indicate p-values >0.05, <0.05, <0.01, <0.001 respectively.

The HSV-1 tegument protein VP22 blocks activation of the AIM2 inflammasome [22] and, therefore, it is unsurprising that we failed to find a dependence on AIM2. However, it is possible that HSV-1 has evolved multiple mechanisms to alter inflammasome activation. To test this hypothesis, we cultured M0 and M1 MDMs with HSV-1 or UV irradiated HSV-1 (HSV-1/UV). Interestingly, HSV-1/UV exposure did lead to IL-18 production in M0 macrophages (**Fig 3A**). This result suggests that M0 macrophages are capable of inflammasome formation in response to HSV-1, but that a viral factor that is produced during the replication cycle (such as VP22) inhibits this activation. When added to M1 macrophages, HSV-1/UV led to significantly increased IL-18 production compared to HSV-1 (**Fig 3B**). At four hours post infection, little IL-18 was detectable in supernatants from M1 macrophages, while at eight hours post infection, IL-18 was significantly higher in M1 macrophages infected with HSV-1 compared to those infected with HSV-1/UV (**Fig 3C**). These data suggest that when macrophages are skewed toward an inflammatory state with IFNγ, a cellular factor is altered that counteracts the inhibitory mechanism(s) of the virus in M0 macrophages. However, replication of the virus does continue to lead to some downregulation of inflammasome activation in IFNγ-treated macrophages because HSV-1/UV led to increased IL-18 production versus HSV-1, albeit not until 24 hours post infection. One explanation for this phenomenon is that UV-irradiating the virus eliminates sufficient production of VP22 such that AIM2 is able to sense the viral DNA and trigger inflammasome formation in M0s. Whereas the replication competent virus inhibits AIM2 via VP22, the M0s lack additional factor(s) required to trigger inflammasome signaling in response to HSV-1. After skewing with IFNγ, HSV-1 infection leads to inflammasome formation through a non-AIM2 dependent mechanism and HSV-1/UV is able to trigger inflammasome signaling through both AIM2 dependent and non-AIM2 dependent mechanisms.

**Fig 4:**
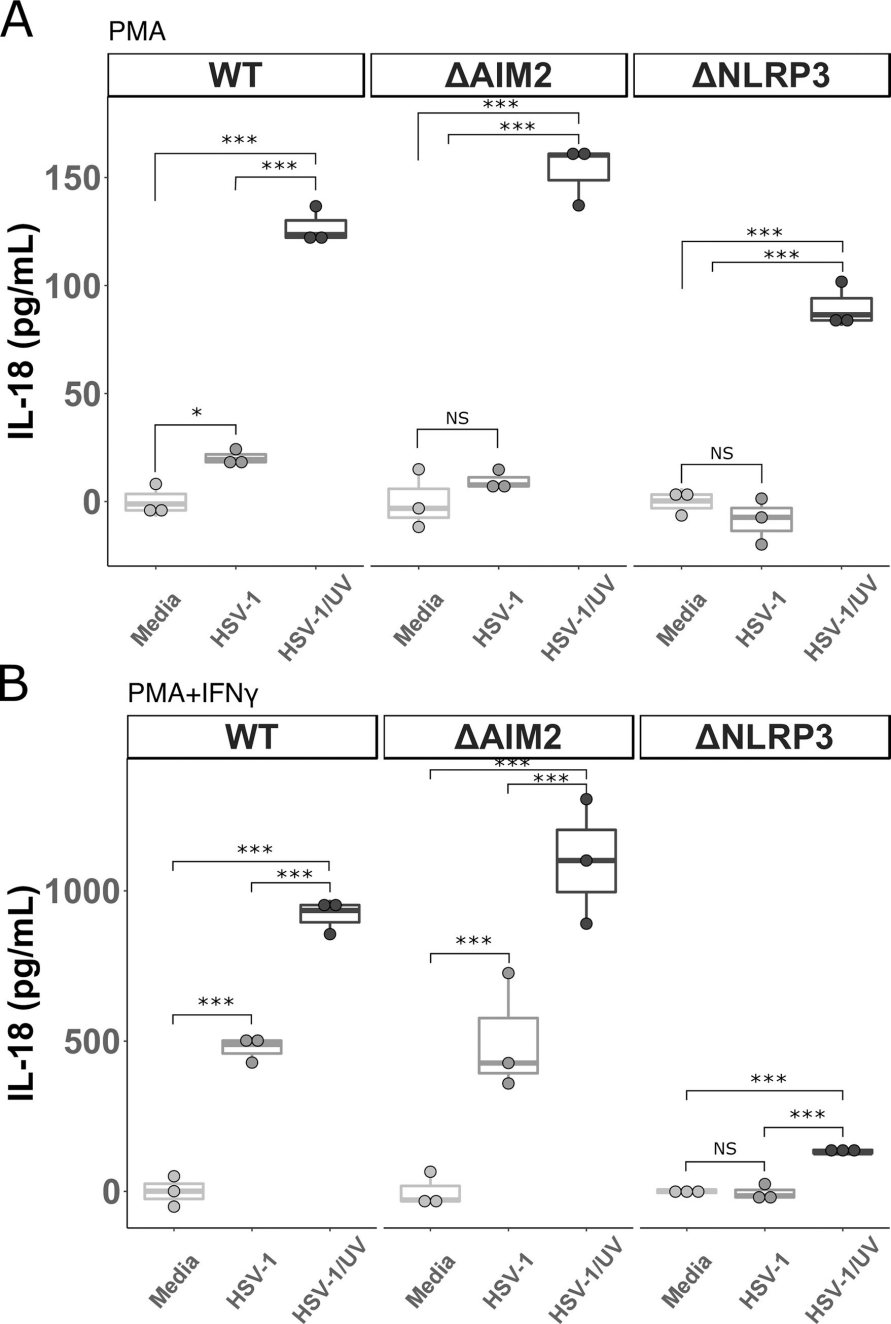
IL-18 produced by THP-1 cells after infection with UV-irradiated HSV-1. **A.** THP-1 cells were stimulated overnight with PMA (5 ng/mL) and then incubated with HSV-1, UV irradiated HSV-1 (HSV-1/UV), or media for 24 hours. Cell culture supernatants were collected and IL-18 was measured via ELISA. **B.** THP-1 cells were stimulated with PMA (5 ng/mL) and then with IFN0γ (25 ng/mL) the following day for 24 hours prior to incubation with HSV-1, UV irradiated HSV-1 (HSV-1/UV), or media alone for 24 hours. Cell culture supernatants were collected and IL-18 was measured via ELISA. ΔHUMCYC cells are labeled as “WT.” Differences between indicated conditions within a cell type were determined by a one-way ANOVA with Tukey HSD post-hoc analysis. NS, *,**,*** indicate p-values >0.05, <0.05, <0.01, <0.001 respectively.

To determine if HSV-1 replication in macrophages results in inhibition of any non-AIM2 inflammasome proteins, we tested HSV-1/UV infection of the THP-1 cells lacking AIM2 and NLRP3 and compared them to WT THP-1 cells. In order to more closely replicate the MDM model with IFNγ stimulation, in this experiment the cells were stimulated with PMA and then either infected directly or stimulated with IFNγ for an additional 24hrs and then infected (**Fig 4**). Similar to what was seen in the MDM model, the WT, ΔAIM2, and ΔNLRP3 cells treated with PMA alone produced more IL-18 in response to HSV-1/UV than in response to replication competent HSV-1 (**Fig 4A**). Interestingly, after the addition of IFNγ, HSV-1 infection led to significant IL-18 production in the WT and ΔAIM2 cells, with even greater IL-18 produced with exposure to HSV-1/UV. Again, the ΔNLRP3 cells did not produce IL-18 in response to HSV-1, but did produce a modest, but statistically significant, amount of IL-18 after infection with HSV-1/UV (**Fig 4B**). These data confirm our findings in the MDMs that HSV-1 infection of unstimulated macrophages does not lead to inflammasome activation. Further, they support the hypothesis that replication competent HSV-1 is capable of decreasing both AIM2 and NLRP3 dependent inflammasome activation because UV irradiating the virus led to significant increases in IL-18 release in both the ΔAIM2 and ΔNLRP3 lines at 24 hours post-infection. IFI16 has been reported to interact with ASC in response to HSV-1 infection and Kaposi's sarcoma-associated herpesvirus (KSHV) in HFF cells early during infection, and the HSV-1 immediate early protein ICPO is known to downregulate IFI16 [41,44,45]. Therefore, we examined IL-18 production in THP-1 cells at 4 and 8 hours post-infection with HSV-1. Similar to what was observed at 24 hours post-infection, HSV-1 infection of the WT, ΔAIM2, and ΔIFI16 THP-1 cells led to significant IL-18 production. However, infection of ΔNLRP3, Δcaspase-1, and ΔASC cells resulted in minimal production of IL-18 not significantly different from mock infection (**S2C and S2D Fig**). This indicates that in proinflammatory macrophages, IFI16 and AIM2 are dispensable for HSV-1 induced inflammasome activation early during infection. In keeping with previously published reports, IFI16 was not decreased at 4 hours post infection with HSV-1, but IFI16 was decreased after 24 hours of infection. HSV-1/UV did not decrease IFI16 expression (**S2B Fig**). As observed in the MDM model, infection with HSV-1/UV did not lead to more robust IL-18 production at these earlier time points, which supports the hypothesis that HSV-1 has evolved mechanisms that require de novo viral protein translation to inhibit inflammasome signaling.

**Fig 5:**
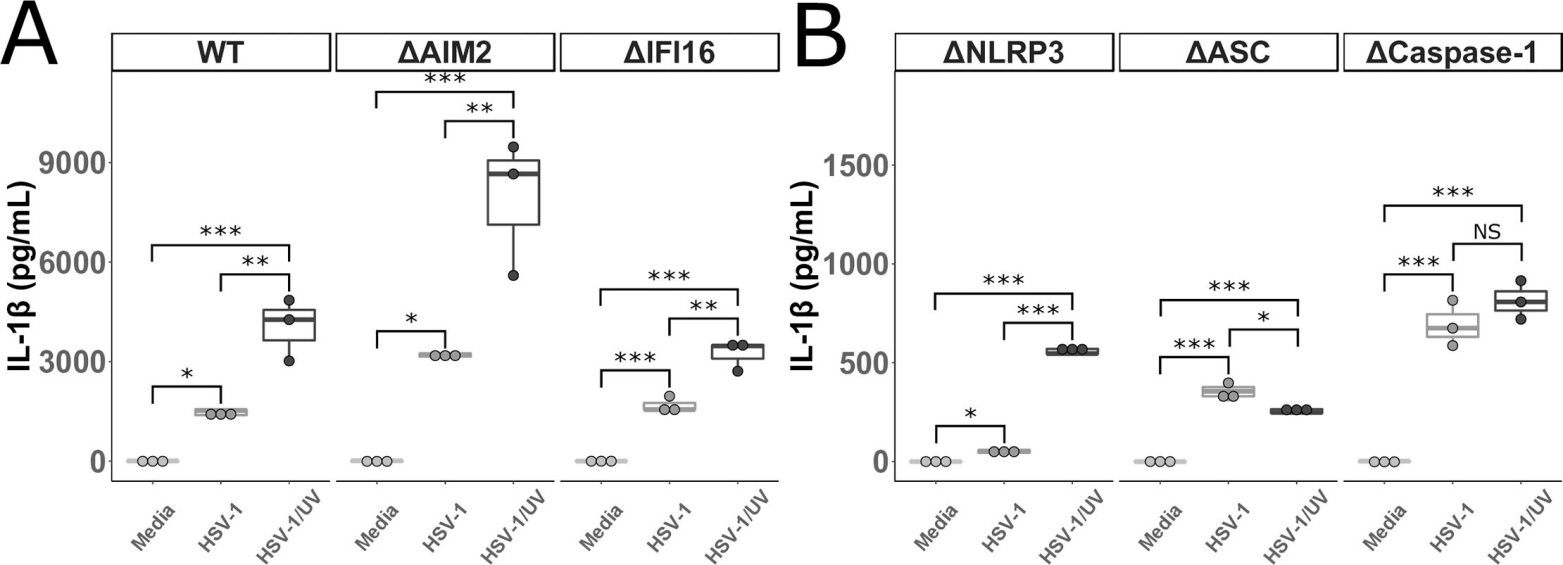
IL-1β produced by THP-1 cells after infection with UV-irradiated HSV-1. **A and B.** THP-1 cells were stimulated with PMA (5 ng/mL) and then with IFNγ (25 ng/mL) the following day for 24 hours prior to incubation with HSV-1, UV irradiated HSV-1 (HSV-1/UV), or media alone for 24 hours. Cell culture supernatants were collected and IL-1β was measured. ΔHUMCYC cells are labeled as “WT.” Differences between indicated conditions within a cell type were determined by a one-way ANOVA with Tukey HSD post-hoc analysis. NS, *,**,*** indicate p-values >0.05, <0.05, <0.01, <0.001 respectively.

IL-1β was also measured in the supernatants from the IFNγ treated THP-1 cells infected with HSV-1 or HSV-1/UV. Similar to IL-18, significant amounts of IL-1β were produced by WT, ΔAIM2, and ΔIFI16 THP-1 cells infected with HSV-1, and HSV-1/UV led to even greater amounts of IL-1β (**Fig 5A**). Unlike IL-18, IL-1β was detected in supernatants from ΔNLRP3, ΔASC, Δcaspase-1 THP-1 cells infected with HSV-1 and HSV-1/UV (**Fig 5B**). This is consistent with what was observed in the MDMs treated with VX-765 and confirms that HSV-1 is capable of inducing IL-1β release in both canonical and non-canonical inflammasome pathways in macrophages.

## Discussion

In this study, we demonstrate for the first time that HSV-1 induces IL-18 production and activation of inflammasomes in primary human macrophages stimulated with IFNγ through a caspase-1 dependent process. UV irradiating the virus prior to infection also leads to IL-18 production in unstimulated primary macrophages, but replication competent HSV-1 does not result in IL-18 release without pre-treatment with IFNγ. Furthermore, using THP-1 cell lines, we show that HSV-1 induced canonical inflammasome activation is dependent on NLRP3, ASC, and caspase-1. By comparing HSV-1 and HSV-1/UV in these THP-1 cells, we also provide evidence that HSV-1 is capable of decreasing inflammasome activation through AIM2 and NLRP3 dependent mechanisms. Unlike IL-18, we found that macrophages are capable of producing IL-1β in a non-canonical, NLRP3, ASC, and caspase-1 independent manner in response to HSV-1. Finally, activation of the inflammasome in macrophages does not prevent viral replication as measured by plaque assay on macrophage culture supernatants 24 hours after infection with HSV-1.

Our data indicate that a cellular factor is altered in macrophages stimulated with IFNγ that allows for activation of the inflammasome after HSV-1 infection. Multiple cellular pathways and metabolic programs are modulated in macrophages exposed to IFNγ [28,29]. While IFNγ is generally accepted as a proinflammatory stimulus, some inflammatory pathways, including IL-1β production, can be blunted after IFNγ stimulation in murine, bone-marrow derived macrophages [46]. While we do not know what factor is altered, it is unlikely that it is NLRP3 itself because prior studies have demonstrated no significant increases in NLRP3 expression in macrophages after skewing with IFNγ [24,47].

Although a previous study suggested that HSV-1 infection of primary human macrophages does not lead to inflammasome activation [48], the macrophages in that study were only stimulated with the TLR2 agonist Pam_3_Cys and not IFNγ. Our data in M0-like MDMs showing that HSV-1 infection failed to induce IL-18 secretion are in agreement with this previous study. HSV-1 has been reported to stimulate multiple inflammasome adapter proteins in non-macrophage cell types. In HFFs, HSV-1 was shown to stimulate inflammasome activation through NLRP3 and IFI16 [41] and in keratinocytes it was suggested that HSV-1 activates inflammasomes via NLRP3, IFI16, and AIM2 [40]. However, in our study we found that HSV-1 inflammasome activation in proinflammatory macrophages is dependent on NLRP3, but not IFI16 or AIM2. It is possible that different cell types utilize different inflammasome signaling mechanisms in response to pathogens and that HSV-1 does activate the inflammasome through IFI16 or AIM2 in non-macrophage cells. Moreover, the immediate early HSV-1 protein, ICP0, is known to degrade IFI16, which may explain why IFI16 does not play a role in inflammasome activation in this model using proinflammatory macrophages [41,45]. Indeed, we observed decreased IFI16 in THP-1 cells infected with HSV-1 for 24 hours, an effect negated by UV irradiation. Furthermore, the lack of dependence on IFI16 and AIM2 does not rule out a role for other dsDNA sensors in HSV-1 inflammasome activation. The NLRP3 inflammasome can be triggered by a STING mediated mechanism in myeloid cells [49]. Importantly, IFI16, cGAS-STING, and other dsDNA sensors have critical roles in innate signaling particularly in triggering the type I interferon response (reviewed in [50]), and sensing by cGAS has also been shown to prime cells for inflammasome activation [51]. Innate sensing of DNA viruses is quite complex, and multiple sensors can interact, as is the case of IFI16 and cGAS-STING in HFFs [52]. Therefore, it is possible there is redundancy in these dsDNA sensing molecules and one or more is sensing the viral nucleic acid upstream of NLRP3. A recent study in which wild-type THP-1 cells were infected with several strains of HSV-1 showed that more virulent strains of HSV-1 induced more mature IL-18 (measured by western blot) production and that multiple inflammasome adapter proteins were upregulated after HSV-1 infection, including NLRP3, NLRP6, NLRP12, and IFI16 [18]. However, it is known that HSV-1 infection leads to upregulation of multiple proinflammatory genes. Therefore, increased expression of these inflammasome related proteins does not necessarily indicate that inflammasome activation by HSV-1 is taking place through these adapters [48,50,53]. Although CMV, a closely related herpesvirus, was recently discovered to activate the inflammasome through AIM2 [43], initial studies on HSV-1 inflammasome activation in macrophages did not find a dependence on AIM2 [21]. This finding was explained by the discovery that VP22 specifically inhibits the interaction between AIM2 and the HSV-1 genome [22]. In agreement with these studies, our current investigation found that AIM2 was not required for HSV-1 to activate the inflammasome in THP-1 cells. Moreover, UV-irradiated HSV-1 led to more IL-18 production at 24 hours post-infection, suggesting more robust inflammasome signaling in response to UV-irradiated HSV-1 in both IFNγ stimulated and unstimulated macrophages. UV-irradiated virus is unable to produce de-novo VP22 and therefore the virus is able to activate the inflammasome both through AIM2 and NLRP3. To further support this, the ΔNLRP3 THP-1 cells produced IL-18 in response to UV-irradiated, but not replication competent HSV-1. With irradiation, there is insufficient VP22 present to inhibit AIM2 and thus the macrophages are able to sense the HSV-1 genome via AIM2. Interestingly, UV-irradiating HSV-1 prior to infection also led to a robust increase in IL-18 release in the ΔAIM2 THP-1 cell line compared to WT virus. If the VP22-AIM2 interaction were the only mechanism by which HSV-1 is capable of inhibiting inflammasome activation, we would expect no difference in IL-18 production between ΔAIM2 cells infected with replication competent HSV-1 or UV-irradiated HSV-1 because AIM2 is not present. However, we found that UV-irradiating the virus led to an increase in IL-18 in ΔAIM2 THP-1 cells at 24 hours post infection, suggesting that the virus has evolved other mechanisms to inhibit inflammasome activation in macrophages that are not AIM2 dependent. This is in agreement with a previous report that HSV-1 interferes with NLRP3-ASC interaction in HFFs [41]. Having multiple mechanisms of evasion highlights the importance of inflammasome activation in macrophages to control HSV-1.

The primary limitation of our study is that it was restricted to primary macrophages and macrophage-like cell lines. As discussed, our data support that HSV-1 is capable of activating more than one inflammasome signaling adapter and the signaling pathway may differ depending on the cell type studied. Therefore, we cannot draw conclusions regarding the interaction between HSV-1 and IFI16 or other inflammasome related proteins in all cell types the virus is capable of infecting. However, macrophages are a crucial cell type in inflammasome activation and HSV-1 control in murine models, prompting our focus on this cell type. Additionally, the present studies were centered on human cells and cell lines and we did not investigate these inflammasome proteins in other species or whole animal models. A prior study in mice showed that HSV-1 causes more severe keratitis after corneal infection in NLRP3 KO mice compared to WT [54]. This suggests that regulation of this pathway is central to the delicate balance between viral control and excessive tissue damage.

In summary, we have demonstrated that HSV-1 infection leads to production of IL-18 through canonical caspase-1 inflammasome activation in proinflammatory primary human macrophages. This process is dependent on the inflammasome proteins NLRP3, ASC, and caspase-1. However, IL-1β release in macrophages infected by HSV-1 occurs through both canonical and non-canonical inflammasome activation pathways. Furthermore, our data demonstrate that HSV-1 replication partially inhibits NLRP3 dependent inflammasome activation in human cells.

## Materials and methods

### Cells and viruses

HSV-1 strain KOS was the generous gift of Richard Longnecker (Northwestern University). Virus was propagated in Vero cells (also a gift from Richard Longnecker, Northwestern University) cultured in Dulbecco’s modification of Eagle medium with 1% fetal bovine serum as previously described [55]. Standard plaque titrations to determine viral titers were performed on confluent monolayers of Vero cells. For UV inactivation, the inoculum was dispensed in a sterile basin in a biosafety cabinet with a UV lamp source (Sankyo Denki G30T8) and irradiated for 30 minutes. Virus inactivation was confirmed by standard plaque assay. Titer decrease of ≥ 10^6^ PFU/mL was considered successful. Cells were incubated at 37°C and 5% CO_2_ unless otherwise stated. Vero cells were maintained in Dulbecco’s modification of Eagle medium with 10% fetal bovine serum and penicillin/streptomycin (50 U/mL, ThermoFisher) (DME). PBMCs were isolated by Ficoll-Hypaque gradient centrifugation. Primary monocytes were magnetically sorted by negative isolation per the manufacturer’s specifications (Miltenyi Biotec, Somerville, Massachusetts) and cultured in RPMI 1640 (Invitrogen, Waltham, Massachusetts) with 10% heat-inactivated fetal bovine serum, Penicillin/Streptomycin, L-glutamine (2mM) and 50 ng/mL of recombinant human M-CSF (R&D Systems, Minneapolis, Minnesota) for 6 to 7 days to differentiate them to macrophages [56]. Adherent macrophages were washed with sterile PBS and then incubated with the non-enzymatic cell disassociation media, CellStripper (Corning, Tewksbury, Massachusetts), for 30 minutes at 37°C and 5% CO_2_ followed by counting, centrifugation at 400g for 5 min, and plating at a density of 3x10^5^ cells/well in a sterile U-bottom 96-well plate (unless otherwise stated). For M1 differentiation, macrophages were cultured overnight in RPMI 1640 with 10% heat-inactivated fetal bovine serum, Penicillin/Streptomycin, L-glutamine (2mM), and IFNγ (25ng/mL) (Peprotech, Rocky Hill, New Jersey) [47]. M0 macrophages were cultured in the same base media, but without IFNγ. The generation of the THP-1 cells was well described previously [43]. THP-1 cells were maintained in RPMI 1640 media, 10% heat-inactivated fetal bovine serum, MEM nonessential amino acids (1:100, Corning, cat# 25–025), Penicillin/Streptomycin, sodium pyruvate, and L-glutamine (2mM) at a density of 5x10^5^ – 2x10^6^ cells/mL. To differentiate into macrophages THP-1 cells were plated at a density of 3x10^5^ cells/well in a sterile U-bottom 96-well plate and stimulated overnight in RPMI 1640 media, 2% heat-inactivated fetal bovine serum, Penicillin/Streptomycin, L-glutamine (2mM), and phorbol 12-myristate 13-acetate (PMA) 100 ng/mL (unless otherwise stated).

### Antibodies

Wild-type and Fc Silent^TM^ neutralizing monoclonal antibodies against HSV-1 gD (clone E317) were purchased from Absolute Antibody (Oxford, UK). Neutralization was determined by a standard plaque reduction assay on Vero cells. Unless otherwise stated they were used at a concentration of 2 μg/mL in experiments.

### Infections and inflammasome activation

Unless otherwise stated, all infections were carried out at a multiplicity of infection (MOI) of 10. For both HSV-1 infection and nigericin stimulation, media were gently aspirated from the cell culture wells containing the indicated cells and replaced with RPMI 1640 media containing 2% heat-inactivated fetal bovine serum (R2) and either HSV-1 (at a MOI of 10), nigericin (5μM) (MilliporeSigma, Burlington, Massachusetts) and LPS (1 μg/mL), or no additional reagents (mock/media control). Twenty-four hours later supernatant was removed and used for downstream assays. To measure viral progeny produced in macrophages (**Fig 1F and Fig 2B**), macrophages were plated in sterile 12-well culture dishes at a density if 5x10^5^ cells/well. Media were aspirated and replaced with HSV-1 strain KOS in R2 and incubated at 37°C for 1 hr. The inoculum was aspirated, cells were washed with sterile phosphate-buffered saline (PBS), washed with a citrate solution (pH 3) to inactivate any viral particles that had not entered, and fresh warm R2 was added back to the cells. 24 hours later cell culture media were harvested and PFU were determined by plaque assay.

### IL-18, TNFa, and IL-1β measurements

Human IL-18, TNFα, and IL-1β were measured with the human IL-18 ELISA Kit (MBL, Woburn, Massachusetts), human TNFα ELISA Kit (ThermoFisher, Waltham, Massachusetts), and IL-1β MesoScale Discovery (Meso Scale Discovery, Gaithersburg MD) electrochemiluminescence assay according to the manufacturers’ instructions using cell culture supernatant at a 1:5 dilution. The lower limit of detection was 12 pg/mL for IL-18, 7.8 pg/mL for TNFα, and 0.05 pg/mL for IL-1β. Data were acquired on a SpectraMax M2 and MESO QuickPlex SQ 120. Results were analyzed using R. Unless otherwise stated, all measurements were normalized to the average of the media control for each experiment.

### Western blots

Unless otherwise stated, THP-1 cells were stimulated with PMA (5 ng/mL) and then with IFNγ (25 ng/mL) the following day for 24 hours prior to stimulation. Cells were lysed on ice using Cell Lysis Buffer (Cell Signaling Technologies, Danvers, MA) supplemented with protease inhibitors (1:100, Cell Signaling Technologies, cat# 5872) and stored at -80°C for western blot analysis. Lysates were run on 4–12% BIS-TRIS gels (ThermoFisher, Waltham, MA) in reducing conditions before transfer to nitrocellulose membranes. Blots were blocked and then incubated overnight with anti-caspase-1 (polyclonal, Cell Signaling technologies), anti-β-actin (BA3R, ThermoFisher), or anti-IFI16 (D8B5T, Cell Signaling Technologies) antibodies as indicated, washed, incubated with an appropriate secondary antibody, developed with ECL (GE, Marlborough, MA), and imaged on a BioRad ChemiDoc XRS+.

### Statistical analysis

Each experiment was repeated at least twice. Statistical analysis was performed on the results of each individual experiment unless otherwise noted in the figure legend.

### Ethics statement

For experiments involving primary human macrophages, deidentified human blood Leuko Paks were obtained from the Anne Arundel Medical Blood Donor Center (Anne Arundel, Maryland, USA). The researchers had no interaction with the donors and did not have any knowledge about them beyond their status as volunteer blood donors. This is considered non-human subjects research by the institutions where the research was conducted and US Department of Health and Human Services guidelines.

## Supporting information

S1 Fig **A.** Primary human MDMs cultured without (M0 left panel) or with IFNγ (M1 right panel) were incubated with nigericin and LPS (Ng+LPS), or media, as outlined in Materials and Methods, for 24 hours. Cell culture supernatants were collected and IL-18 was measured. **B.** MDMs cultured with IFNγ or IFNγ and LPS were either mock infected or infected with HSV-1 for 24 hours. Cell culture supernatants were collected and IL-18 was measured. Differences between groups indicated by brackets were determined by a Student’s *t*-test. NS, *,**,*** indicate p-values >0.05, <0.05, <0.01, <0.001, respectively. In **A**, the M1 condition (right panel) is the combination of two experiments.
(TIF)

S2 Fig **A.** THP-1 cells with the indicated gene disrupted via CRISPR-cas9 (Δ) were stimulated overnight with PMA and then incubated with HSV-1, or media for 24 hours before IL-18 was measured in cell supernatants. ΔHUMCYC cells are labeled as “WT.” Differences between groups indicated by brackets were determined by a Student’s *t*-test. NS, *,**,*** indicate p-values >0.05, <0.05, <0.01, <0.001, respectively. These data are the same data as in **Fig 2D**, but graphed to show similarities between indicated cell types. **B.** Cell lysates from WT THP-1 cells infected with HSV-1 or mock infected (left panel) were probed for IFI16 or β-actin via western blot 4 hours post infection. Cell lysates from WT THP-1 cells infected with UV irradiated HSV-1, HSV-1 or mock infected (right panel) were probed for IFI16 or β-actin via western blot 24 hours post infection. **C and D**. THP-1 cell lines with the indicated gene disrupted by CRISPR-cas9 (Δ) were stimulated with PMA (5 ng/mL) and then with IFNγ (25 ng/mL) the following day for 24 hours prior to incubation with HSV-1, UV irradiated HSV-1 (HSV-1/UV), or media alone for **(C)** 4 hours or **(D)** 8 hours and IL-18 was measured in supernatants.
(TIF)

S3 Fig (PDF)
