# Supplementary figures and images for: Herpes simplex virus type 1 inflammasome activation in proinflammatory human macrophages is dependent on NLRP3, ASC, and caspase-1
Source: PLoS One. 2020 Feb 26;15(2):e0229570. doi: 10.1371/journal.pone.0229570 (PMC7043765; doi:10.1371/journal.pone.0229570)

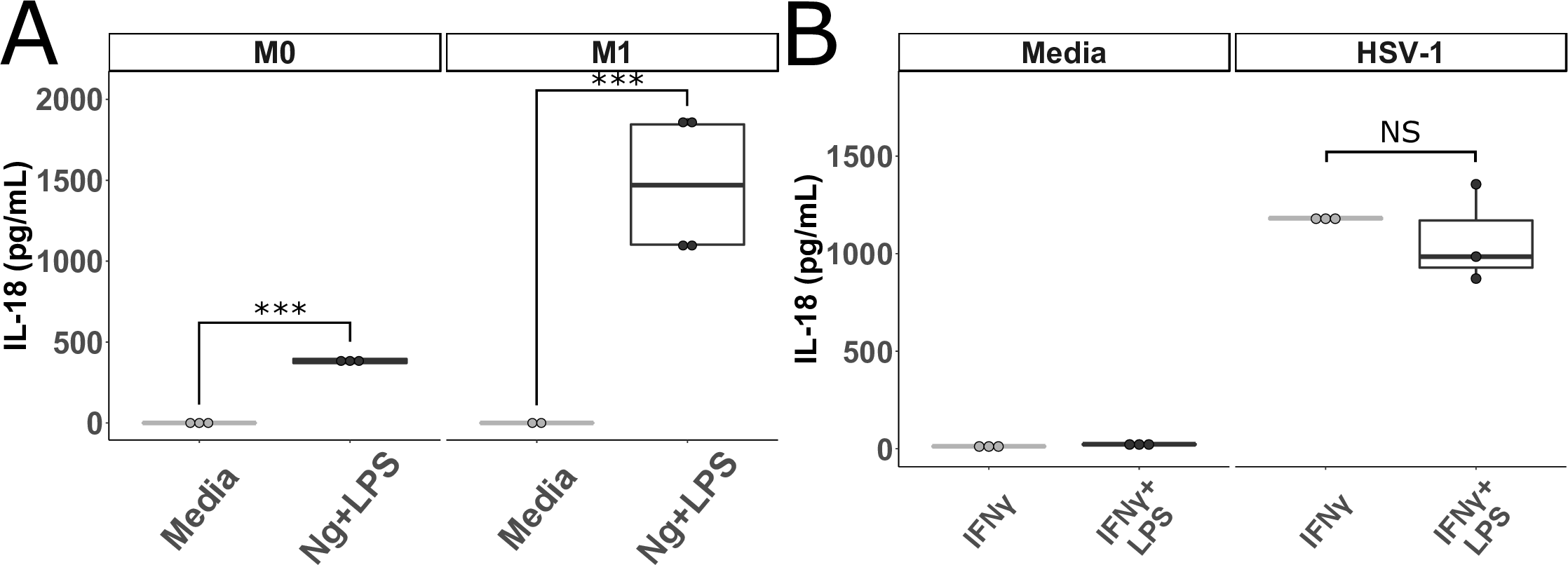

Supplement: S1 Fig — A. Primary human MDMs cultured without (M0 left panel) or with IFNγ (M1 right panel) were incubated with nigericin and LPS (Ng+LPS), or media, as outlined in Materials and Methods, for 24 hours. Cell culture supernatants were collected and IL-18 was measured. B. MDMs cultured with IFNγ or IFNγ and LPS were either mock infected or infected with HSV-1 for 24 hours. Cell culture supernatants were collected and IL-18 was measured. Differences between groups indicated by brackets were determined by a Student’s t-test. NS, *,**,*** indicate p-values >0.05, <0.05, <0.01, <0.001, respectively. In A, the M1 condition (right panel) is the combination of two experiments. (TIF) [file pone.0229570.s001.tif]

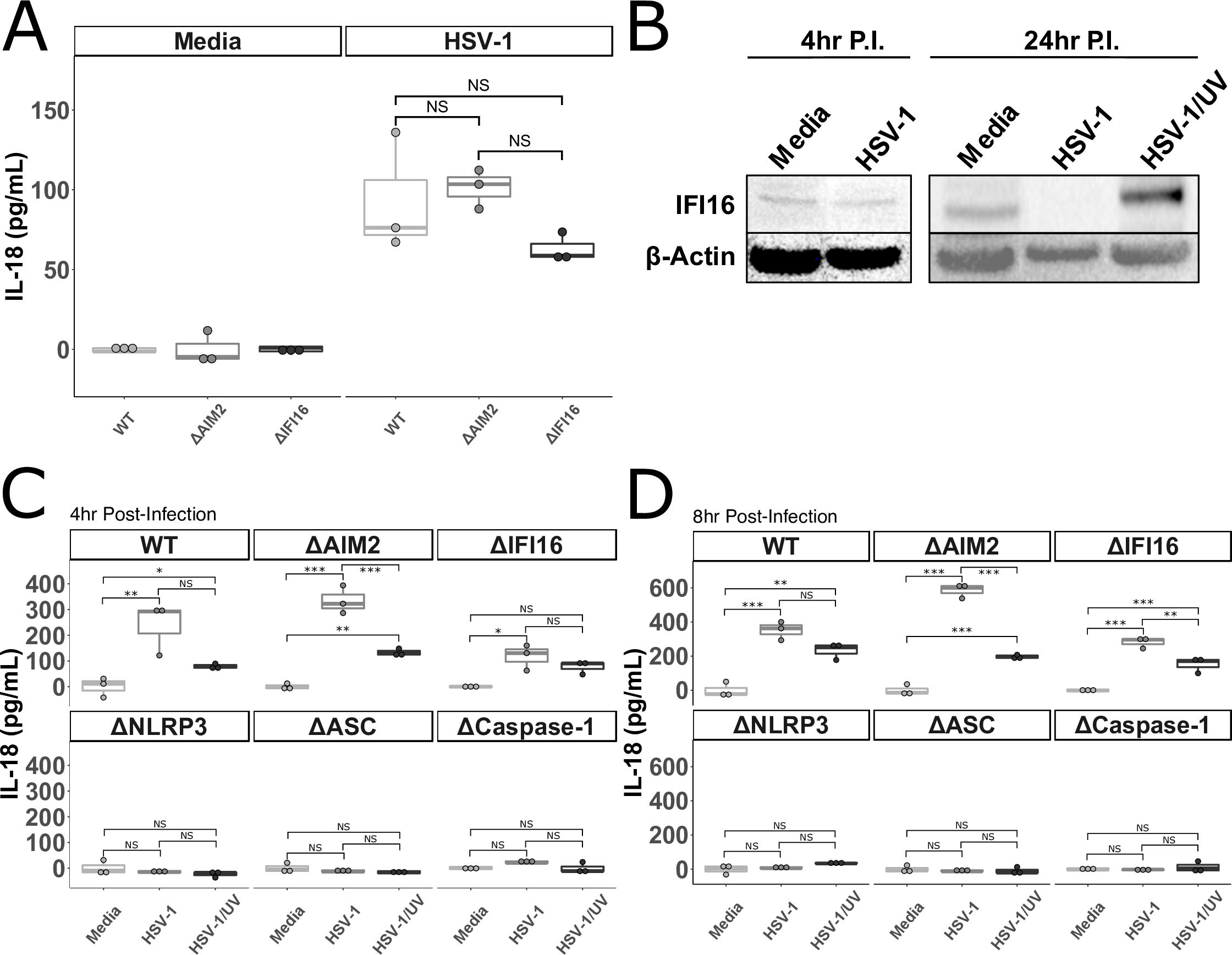

Supplement: S2 Fig — A. THP-1 cells with the indicated gene disrupted via CRISPR-cas9 (Δ) were stimulated overnight with PMA and then incubated with HSV-1, or media for 24 hours before IL-18 was measured in cell supernatants. ΔHUMCYC cells are labeled as “WT.” Differences between groups indicated by brackets were determined by a Student’s t-test. NS, *,**,*** indicate p-values >0.05, <0.05, <0.01, <0.001, respectively. These data are the same data as in Fig 2D, but graphed to show similarities between indicated cell types. B. Cell lysates from WT THP-1 cells infected with HSV-1 or mock infected (left panel) were probed for IFI16 or β-actin via western blot 4 hours post infection. Cell lysates from WT THP-1 cells infected with UV irradiated HSV-1, HSV-1 or mock infected (right panel) were probed for IFI16 or β-actin via western blot 24 hours post infection. C and D. THP-1 cell lines with the indicated gene disrupted by CRISPR-cas9 (Δ) were stimulated with PMA (5 ng/mL) and then with IFNγ (25 ng/mL) the following day for 24 hours prior to incubation with HSV-1, UV irradiated HSV-1 (HSV-1/UV), or media alone for (C) 4 hours or (D) 8 hours and IL-18 was measured in supernatants. (TIF) [file pone.0229570.s002.tif]

A

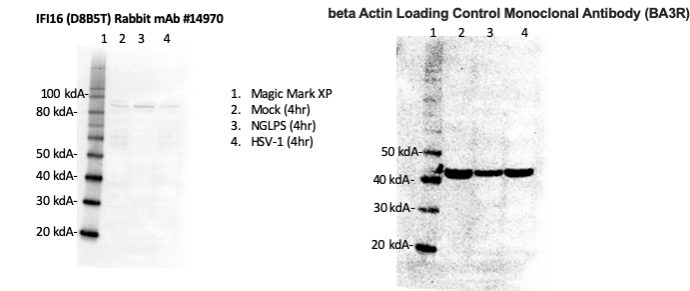

B

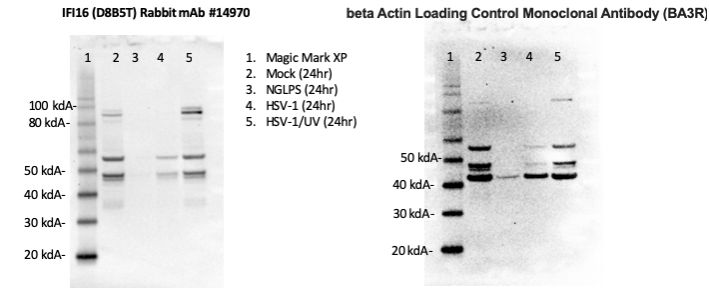

C

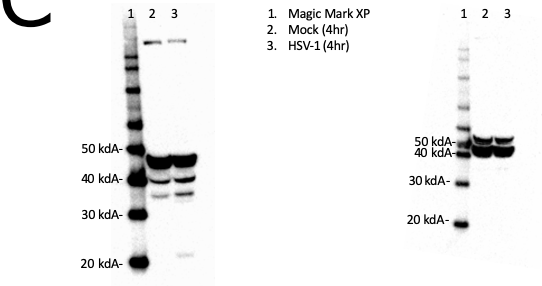

Supplement: S3 Fig — (PDF) [file pone.0229570.s003.pdf]
